# Estimated economic benefits from low-frequency administration of atypical antipsychotics in treatment of schizophrenia: a decision model

**DOI:** 10.1186/1744-859X-11-29

**Published:** 2012-11-16

**Authors:** Nicolas M Furiak, James C Gahn, Robert W Klein, Stephen B Camper, Kent H Summers

**Affiliations:** 1 Medical Decision Modeling Inc, 8909 Purdue Road Suite 550, Indianapolis, IN 46268, USA; 2 Endo Pharmaceuticals, Chadds Ford, PA, USA

## Abstract

The objective of this study was to quantify the direct medical resources used and the corresponding burden of disease in the treatment of patients with schizophrenia. Because low-frequency administration (LFA) of risperidone guarantees adherence during treatment intervals and offers fewer opportunities to discontinue, adherence and persistence were assumed to improve, thereby reducing relapses of major symptoms.

A decision tree model including Markov processes with monthly cycles and a five-year maximum timeframe was constructed. Costs were adapted from the literature and discounted at a 3% annual rate. The population is a demographically homogeneous cohort of patients with schizophrenia, differentiated by initial disease severity (mildly ill, moderately ill, and severely ill). Treatment parameters are estimated using published information for once-daily risperidone standard oral therapy (RIS-SOT) and once-monthly risperidone long-acting injection (RIS-LAI) with LFA therapy characteristics derived from observed study trends. One-year and five-year results are expressed as discounted direct medical costs and mean number of relapses per patient (inpatient, outpatient, total) and are estimated for LFA therapies given at three, six, and nine month intervals.

The one-year results show that LFA therapy every 3 months (LFA-3) ($6,088) is less costly than either RIS-SOT ($10,721) or RIS-LAI ($9,450) with similar trends in the 5-year results. Moreover, the model predicts that LFA-3 vs. RIS-SOT vs. RIS LAI therapy will reduce costly inpatient relapses (0.16 vs. 0.51 vs. 0.41). Extending the interval to six (LFA-6) and nine (LFA-9) months resulted in further reductions in relapse and costs.

Limitations include the fact that LFA therapeutic options are hypothetical and do not yet exist and limited applicability to compare one antipsychotic agent versus another as only risperidone therapy is evaluated. However, study results have quantified the potential health state improvements and potential direct medical cost savings achievable with the development and use of LFA medication delivery technologies.

## Background

Illness relapse remains a primary clinical and economic concern in the treatment of patients with schizophrenia. It is associated with significant direct medical and societal costs [1,2], increased illness symptomology and functional deterioration [3], and a reduced patient [4] and care-giver [5] quality of life. Patients who relapse experience as much as a four-fold increase in costs compared to those who do not relapse [1]. In the US, the direct medical expenditures attributable to relapse exceed $22 billion with the cost of inpatient care being the primary cost contributor [6-8].

Relapse occurrence is a multi-factorial problem. However, suboptimal medication adherence and/or persistence are generally cited as a primary causative factor leading to significant increases in resource utilization [9-11]. Conversely, optimizing medication compliance has been shown to improve symptoms and decrease the frequency and duration of hospital stays [11].

Prolonging the duration of action of an administered agent with a resultant reduction in dosing frequency represents one strategy for improving medication compliance. Several delivery systems have been evaluated for their ability to provide extended drug exposure including extended/sustained-release oral formulations, depot injections, various implant technologies and transdermal patches [12]. At present, two long-acting injectable (LAI) formulations of second generation antipsychotic medications are available in the US for the treatment of schizophrenia. Risperidone long-acting injection [13] is administered every two weeks while paliperidone palmitate extended-release injectable suspension [14] is administered 1x/month. To date, the data regarding the effect of the LAIs on relapse-related hospitalizations is equivocal with some [15,16] but not all [17] reports suggesting a reduction in the frequency and/or duration of relapse-related hospital stays or resource utilization.

Given the generally positive clinical and economic consequences of prolonging the duration of action of antipsychotic agents, it is of interest to examine the potential impact of an even greater prolongation of action on illness relapse rates. Therefore, the objective of this study was to quantify the resources (from a third party payer perspective) used and the corresponding burden of disease due to a projected reduction in relapse frequency resulting from further improvements in medication compliance in patients with schizophrenia.

## Methods

### Overview

**Figure 1:**
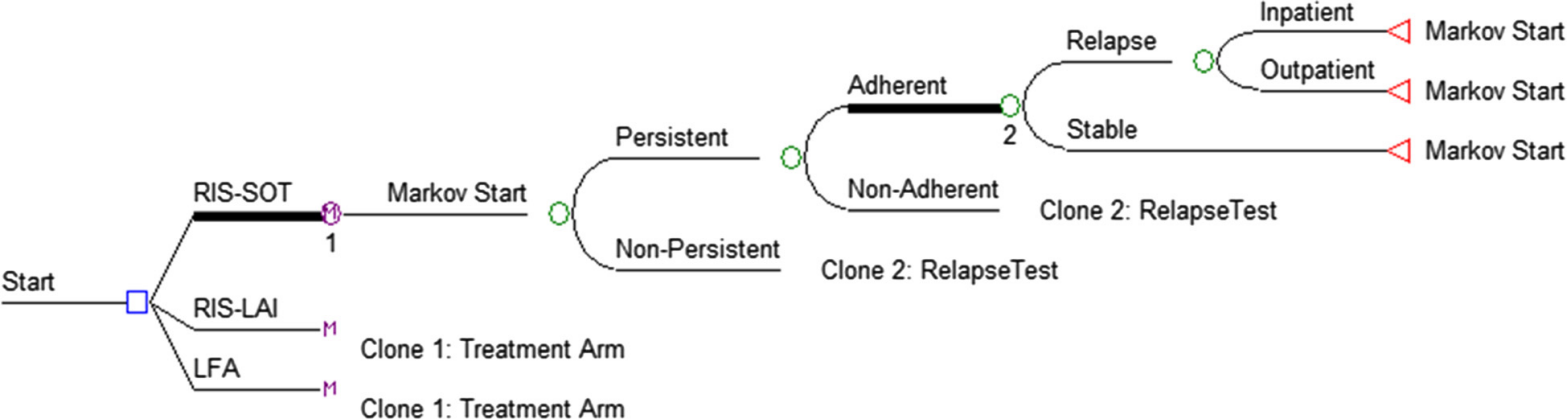
Model structure.

A decision tree model which branched to Markov processes was constructed (Figure 1). The Markov processes had monthly cycles and a five-year timeframe. Costs and quality adjusted life years (QALYs) were discounted at a 3% annual rate. For verification purposes, the model was developed separately in TreeAge 2009 and Microsoft Excel 2010 and predicted results from each platform were compared. Direct medical costs were derived from published studies and applied on a per-setting basis: stable in the community, relapse requiring an inpatient bed, relapse not requiring an inpatient bed.

This study estimates potential cost savings of a hypothetical delivery technology that prolongs the duration of action of an antipsychotic medication thereby permitting low frequency administration (LFA) antipsychotic therapy. Costs include direct clinical resources consumed during routine interaction with healthcare professionals, during relapses occurring in the outpatient setting, and during relapses occurring in the inpatient setting.

Given the abundance of economic analyses for risperidone (RIS) [15,18,19] in the literature, RIS has been chosen as the foundation of therapy for this model in generic standard oral therapy (RIS-SOT) and risperidone long-acting injection (RIS-LAI) formulations. Therefore, the base case model was populated with literature-based rates of adherence and persistence for RIS-SOT and RIS-LAI along with corresponding rates of relapse [20,21].

The following terms have been defined to facilitate the analyses reported. A therapy with a “low frequency of administration” is labeled “LFA” and is assumed to be administered less frequently than currently available treatments administered every 2 weeks. A number appearing next to the LFA label indicates the frequency of administration (i.e. LFA-3 indicates administration every 3 months).“Interval” when referring to therapy administration represents the time between scheduled administrations of therapy. “Routine Care” describes standard, recommended care for all patients with schizophrenia such as regular physician visits and counseling services. “Outpatient relapse” describes an increase in utilization of resources by the patient without requiring an inpatient stay in a hospital bed. “Inpatient relapse” includes increased direct medical resource use in addition to an inpatient bed plus other hospital resources. “Step Down Care” describes a category of care occupied by patients as they transition from an inpatient stay back to routine care in the community.

### Assumptions

The following are the primary assumptions of the model:

1. Simulated patients are a homogeneous cohort of an “average” patient with schizophrenia.

2. The baseline efficacy of each scenario is the same given that a patient is adherent to therapy.

3. Adverse events are assumed to be the same across all administration modes, therefore adherence and persistence are driven by administration frequency only.

4. Patients incur costs corresponding to “Routine Care” at all times regardless of relapse status.

### Model parameters

#### Adherence

Medication adherence is defined as “the extent to which a patient acts in accordance with the prescribed interval and dose of a dosing regimen.” [22] For this study, adherence values were taken from the literature for the RIS-SOT and RIS-LAI products [21]. The base case assumption is that patients will, by definition, be 100% adherent to a therapy that is administered once and lasts for an extended period when using the Less Frequent Administration options.

#### Persistence

Medication persistence is defined as “the duration of the time from initiation to discontinuation of therapy.” [22] Because schizophrenia is a chronic illness and pharmacological therapy is a fundamental component of ongoing care, we operationalized persistence as the proportion of patients who discontinued their medications. Specifically, persistence is estimated using reported rates of discontinuation in CATIE phase I for risperidone [20]. Of the four mutually exclusive reasons for discontinuation reported in CATIE phase I, this study relies on all but the adverse event rate, given that the base compound is the same for all scenarios (RIS) regardless of administration method (oral vs. injection vs. LFA).

Consequently, the rates of persistence differ by those reported in CATIE phase I [20] in the following manner:

D_AC_ = risperidone “All Cause” discontinuation reported in CATIE phase I

D_AE_ = risperidone discontinuation due to “Adverse Events” reported in CATIE phase I

D_PD_ = risperidone discontinuation due to the “Patient’s Decision” reported in CATIE phase I

RIS-SOT_PERS_ = 1.0 – (D_AC_ – D_AE_)

LFA_PERS_ = 1.0 – D_PD_

It is assumed that as the frequency of administration decreases (at the same effectiveness), the more likely it would be that a patient would discontinue based solely on their decision. Therefore, the discontinuation rate for an LFA therapy is assumed to be equal to the patient-decision rate from CATIE [20].

#### Relapse rates

Rates of relapse given adherence for all treatments are based on a published risperidone model [15] with a fixed proportion occurring in the inpatient setting derived from the same model [15]. A complete list of base case inputs is shown in Table 1.

**Table 1:** Base case input parameters

**Base Case Modifiable Inputs**				
	**LFA**	**RIS-LAI**	**RIS-SOT**	**Source**
Adherence	1.0*	0.85*	0.65**	** [21]
Annual Discontinuation (Persistence)	0.30	0.50	0.64	[20,23]
**Annual Relapse Rates**				
When Adherent	10.10%			[15]
When Non-adherent	78.70%			
Proportion of Relapses Inpatient	0.53			
**Utilities**				
	**While Stable**	**In Inpatient Relapse**	**In Outpatient Relapse**	
Adherent	0.88	0.53	0.74	[24]
Non-adherent	0.75	0.42	0.63	
**Execution**				
Model Timeframe	5 years			N/A
Cycle Length	1.0 months			
Discount rate (costs)	3%			

#### Resource use

Nicholl et al [19] published a database analysis comparing the resource use between newly diagnosed schizophrenia patients and chronic patients. Most of the unit costs of resource components found in input Table 2 were derived using Nicholl et al [19]. Inpatient and outpatient costs were derived using weighted averages of the new and chronic patients. (Table 2) There was complete overlap of the categories included in the inpatient and outpatient costs in Nicholl et al. [19] which allowed for an approximation of the per unit cost for most resources. Once derived, total mean expenditures for a category were then divided by the derived per unit cost to populate the resource used. When categories between inpatient and outpatient resources did not overlap, the unit costs and number of units were derived using inpatient resources consumed for recently diagnosed patients compared to chronic patients.

**Table 2:** Resource units and costs

**Resource Use Input Table **[19]				
**Resource**	**Cost**	**Units per Outpatient Relapse**	**Units per Inpatient Relapse**	**Routine Care - Annually**
Room and Board	$751 [25]	0.0	10.8 [25]	0.0
Medications	$257	0.0	1.0	0.0
Step down care	$40 [26]	0.0	30.0*	0.0
Lab Tests	$26	1.0	8.0	13.0
ER	$425	1.0	1.0	0.0
Intensive Care	$194	0.0	1.0	0.0
Devices	$71	0.0	1.0	0.5
Psychotherapy	$110	0.0	2.0	13.5
Physician Visits	$70	0.0	0.0	5.0
Other While Stable	$1,701	0.0	0.0	1.0
Other While Inpatient Relapse	$2,379	0.0	1.0	0.0

*All values from Nicholl et al.*[19]except where noted; *= assumption. All costs updated to 2011 costs using Bureau of Labor and Statistics Medical component of the CPI[27].

The values in Table 2 that were derived from sources other than Nicholl et al. are cost per day of hospitalization, the number of days per inpatient stay, and the added cost of step-down care after inpatient relapse. Cost of hospitalization and number of days were derived from 2009 AHRQ HCUP data based on using a primary diagnosis of “Schizophrenia and other psychotic disorders” [25]. Step-down care as listed in Table 2 is assumed to take place for one month at $40 per day based upon a study of community mental health treatment services in 5 cities across the U.S. [26].

## Results

### Base case

Base case results are presented in Table 3 and Table 4 for one-year and five-year timeframes, respectively. The one-year results show that LFA (3 month) is less costly than either RIS-SOT or RIS-LAI. Moreover, the model predicts that LFA therapy will reduce inpatient and total relapses in the first year compared to both RIS-SOT and RIS-LAI therapies. These results suggest that cost offsets due to reduction of inpatient relapses for LFA potentially take effect in year one. The five-year base case results comparing RIS-SOT, RIS-LAI, and LFA are shown in Table 4. Similar to the one-year results, increasing adherence and persistence through LFA therapy leads to lower total costs and an appreciable reduction in relapses over 5 years.

**Table 3:** One-year base case model results (Mean values of the modeled population)

		**RIS-SOT**	**RIS-LAI**	**LFA −3**	**LFA −6**	**LFA −9**
**Direct Medical Costs (Discounted)**	**Routine Care Costs**	$4,013	$4,013	$4,013	$4,013	$4,013
	**Inpatient Costs**	$6,508	$5,276	$2,013	$1,642	$1,398
	**Outpatient Costs**	$199	$161	$62	$50	$43
	**Total Direct Medical Costs**	$10,721	$9,450	$6,088	$5,706	$5,454
**Health States**	**Inpatient Relapses**	0.51	0.41	0.16	0.13	0.11
	**Outpatient Relapses**	0.45	0.36	0.14	0.11	0.10
	**Total Relapses**	0.96	0.77	0.30	0.24	0.21

**Table 4:** Five-year base case model results (Mean values of the modeled population)

		**RIS-SOT**	**RIS-LAI**	**LFA - 3**	**LFA −6**	**LFA −9**
**Direct Medical Costs (Discounted)**	**Routine Care Costs**	$18,326	$18,326	$18,326	$18,326	$18,326
	**Inpatient Costs**	$42,419	$38,721	$26,241	$25,251	$24,261
	**Outpatient Costs**	$1,299	$1,185	$803	$773	$743
	**Total Direct Medical Costs**	$62,043	$58,232	$45,370	$44,349	$43,329
**Health States**	**Inpatient Relapses**	3.52	3.22	2.20	2.12	2.04
	**Outpatient Relapses**	3.12	2.85	1.95	1.88	1.81
	**Total Relapses**	6.63	6.07	4.15	4.00	3.85

### Sensitivity analysis

One way sensitivity analysis was performed on variables in the model by varying base case parameters by ±50%. Major drivers of costs identified were the relapse rate when non-adherent, the cost per inpatient relapse, and the proportion of relapses requiring an inpatient stay (data not shown).

Sensitivity analysis on administration frequency was performed to quantify the impact of decreasing administration frequency on health and economic outcomes. In general, the trend of lower costs with fewer relapses observed in the base case continued as administration frequency decreased.

## Discussion

The objective of this study was to predict the health and economic impact of employing technologies that improve medication compliance by extending drug delivery and decreasing frequency of administration. The study was based on the hypothesis that improving medication compliance will lead to a reduction in illness relapse and the costs associated with treating the relapses. Modeled and clinical support for this hypothesis exists in the published literature [6,9,15,16,21,23,28]. The model used for the presented analysis implements information for risperidone available in the public domain as a basis for forward-thinking analysis. The results estimate the value of seeking opportunities to reduce illness relapse in this patient population.

### Health states

Most published data support the hypothesis that prolonging the duration of action of an antipsychotic medication (with a resultant reduction in dosing frequency) is associated with a reduction in schizophrenia symptom exacerbation compared to standard oral therapy [15,16,21]. Over one year of treatment, the bi-weekly injectable formulation of risperidone reduced the number of relapses per person by 0.2 while, at five years, 0.56 relapses were avoided. The one year reduction in the present analysis is somewhat less than the prediction of 0.6 relapses avoided in a model published by Edwards, et al. [15] and the precise explanation for the difference is unknown. However, the Edwards model predicted a greater number of relapses for both the standard oral therapy and long-acting injectable groups which may have been a contributing factor. Conversely, the five year projection in our model of 0.56 relapses avoided is consistent with data presented by multiple models [21].

At present, there are no long-acting formulations of antipsychotic therapy for schizophrenia with dosing frequencies that extend beyond one month. Moreover, there are no long-acting formulations of risperidone that have dosing frequencies of greater than two weeks. Nonetheless, given the application of existing technologies for prolonging the duration of action of pharmaceutical agents for up to one year in other therapeutic areas, it was of interest to estimate the potentially beneficial effects of applying these technologies to the treatment of schizophrenia. Extending the duration of action to three months was associated with further reductions in the number of relapses compared with both standard, daily risperidone therapy (RIS-SOT) as well as the currently available risperidone long-acting injection (RIS-LAI). Further improvements were seen when the duration of action was extended to six and nine months. Quantified estimates of reductions in relapse produced in this study represent the value of the study methodology and provide evidence supporting the potential therapeutic benefit of extending the duration of action of antipsychotic medications beyond that which is currently available.

### Costs

Because relapse is a primary cost driver in the management of schizophrenia, it is anticipated that a reduction in relapses would be associated with corresponding reductions in the cost of treatment. This association has been reported in the literature [8,28] and is confirmed in the current model. At one year, total direct medical costs were greatest for daily, standard therapy and decreased with increasing duration of action.

In our analysis, improving medication compliance by prolonging duration of action and reducing administration frequency from daily to once every two weeks resulted in a reduction in direct medical costs of almost $1300/patient at the end of one year. The cost reduction seen when comparing the Less Frequent Administration (LFA) options (> 3 months) to RIS-SOT was even more substantial, resulting in cost reductions ranging from $4600 to $5300 per patient. The LFA options also demonstrated cost reductions versus the bi-weekly risperidone injection with the 3-, 6-, and 9-month alternatives reducing costs by $3300/patient, $3700/patient, and $4000/patient, respectively. Importantly, most of the reduction in direct medical costs was produced via a reduction in the costs associated with inpatient care. A sensitivity analysis confirmed the central contribution of relapses requiring inpatient hospitalization to direct medical costs associated with managing schizophrenia.

Additional calculations (data not shown) estimating the societal burden of disease from the base case model were performed using US population data from the 2010 census [29] and the prevalence of schizophrenia data from the National Institute of Mental Health [30]. The incremental difference between RIS-SOT and RIS-LAI was estimated to be a savings of $4.3 billion; extending the frequency of administration to nine months resulted in estimated savings of $17.8 billion compared to RIS-SOT. Base case total burden was found to be consistent with previously published estimates [7].

In this study, we were limited by the fact that risperidone-based therapeutic options with dosing frequencies of greater than two weeks do not exist. While this limitation may have impacted our assumptions concerning the persistence of therapy with the LFA options, it should not have influenced the adherence levels. Assuming that less frequent dosing will be accomplished via utilization of a long-acting injection or implantation of a drug or drug-device combination, once administered, adherence will be 100% for the identified dosing period. The fact that these longer duration alternatives do not exist in schizophrenia also impacts the total absolute costs and cost differentials as we were not able to include “cost of therapy” in our total direct medical cost calculations.

This study is also limited in its applicability to compare the potential benefits of utilizing one antipsychotic agent versus another as we assumed the use of risperidone in all dosing scenarios. However, we believe that restricting our model to one molecule eliminates the potential confounding that might occur as a result of differences in safety/tolerability and efficacy that might be seen when two different molecules are compared.

There are many potentially fertile areas for future research including an opportunity to further evaluate both the clinical and economic consequences of improved medication adherence and persistence in patients with schizophrenia. More data is required to determine the most effective combination of drug delivery mechanism and dosing frequency for optimal disease management. From an economic perspective, the societal costs associated with schizophrenia are well-documented. However, it will be of great interest to predict the potential impact of a new treatment option not only on direct medical costs but also on the larger societal burden.

## Conclusion

This study quantifies the impact of medication adherence and persistence on illness relapse occurrences in patients with schizophrenia. In addition, it demonstrates the potential health state improvements and direct medical cost savings that might be realized from the development of medication delivery technologies that prolong the duration of action of antipsychotic agents beyond that which is currently available.

## Competing interests

RWK is a principal of Medical Decision Modeling Inc., and SBC and KHS are employees of Endo Pharmaceuticals

## Authors’ contributions

NMF researched and designed the model, wrote the first draft of the manuscript and implemented all edits as requested by other Author’s, JCG helped design the model and performed the necessary programming, RWK served as a content consulted and manuscript editor, SBC helped design the model and prepare the manuscript, KHS served as a consultant during model design and contributed to the writing of the manuscript. All authors have read and approved the final, submitted manuscript.
